# Severe obstructive colitis treated with repeated colonoscopic decompression

**DOI:** 10.1002/deo2.233

**Published:** 2023-05-16

**Authors:** Junya Arai, Nobumi Suzuki, Yoku Hayakawa, Hiroyuki Matzuzaki, Yuichiro Yokoyama, Tomonori Aoki, Rei Ishibashi, Nariaki Odawara, Sozaburo Ihara, Yosuke Tsuji, Soichiro Ishihara, Mitsuhiro Fujishiro

**Affiliations:** ^1^ Department of Gastroenterology Graduate School of Medicine University of Tokyo Tokyo Japan; ^2^ Department of Surgical Oncology Graduate School of medicine University of Tokyo Tokyo Japan

**Keywords:** black colon, bowel irrigation solution, colonoscopic decompression, diverticulosis, obstructive colitis

## Abstract

Obstructive colitis is an acute condition caused by colorectal strictures and requires a combination of therapeutic strategies, including surgery, endoscopic interventions, and medications. Here, we describe the case of a 69‐year‐old man who developed severe obstructive colitis owing to diverticular stenosis of the sigmoid colon. We immediately performed endoscopic decompression to avoid perforation. The mucosa of the dilated colon appeared black, suggesting severe ischemia. We considered surgical total colectomy owing to the extensive colitis. However, considering the invasiveness of the emergent surgery, we adopted a conservative approach as enhanced computed tomography demonstrated colonic dilation with maintained blood flow in the deeper layer of the colonic wall and no signs of colonic necrosis, such as peritoneal irritation sign or elevation of deviation enzymes, were observed. Moreover, the patient preferred a conservative approach, and surgeons in our team agreed with this conservative approach. While relapses of colonic dilation occurred several times, antibiotic treatment and repeated endoscopic decompression successfully suppressed the dilation and systemic inflammation. The colonic mucosa healed gradually, and we performed a colostomy without resecting a large portion of the colorectum. In conclusion, severe obstructive colitis with maintained blood flow can be treated with endoscopic decompression instead of emergent resection for a wide portion of the colorectum. Moreover, endoscopic images of improved colitic mucosa obtained through repeated colorectal procedures are rare and noteworthy.

## INTRODUCTION

Obstructive colitis is an acute condition caused by colorectal strictures, mainly owing to colorectal cancers, sigmoid volvulus, and diverticulosis.^1^, ^2^, ^3^, ^4^, ^5^ This colitis type can be severe and sometimes fatal. Therefore, a combination of therapeutic strategies, including surgery, endoscopic interventions, and medications, should be carefully considered.

Here, we present a patient with severe obstructive colitis who was treated with repeated endoscopic decompression. Eventually, a colostomy was performed without resecting a large portion of the colorectum.

## CASE REPORT

A 69‐year‐old man was admitted to our department for an endoscopic colon polypectomy. He had a history of cigarette smoking and alcohol consumption. He had several histories of diverticular bleeding and diverticulitis. Also, he had received endoscopic submucosal dissection for gastric cancer and percutaneous coronary intervention for ischemic heart disease. His height, weight, and body mass index were 169 cm, 73.5 kg, and 25.7 kg/m^2^, respectively.

**FIGURE 1:**
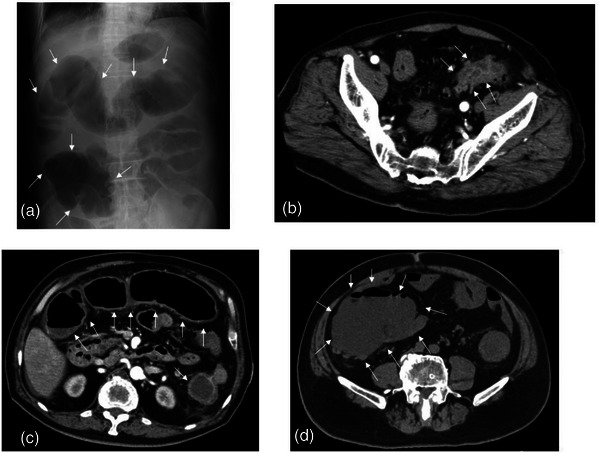
X‐ray and computed tomography images of obstructive colitis. (a) An X‐ray image showing severe colonic dilation from the cecum to the transverse colon. (b–d) Computed tomography images. (b) Obstruction owing to diverticulosis of the sigmoid colon. (c) Enhanced computed tomography image showing maintained blood flow to the colonic wall. (d) Severe dilation of the cecum (>10 cm).

**FIGURE 2:**
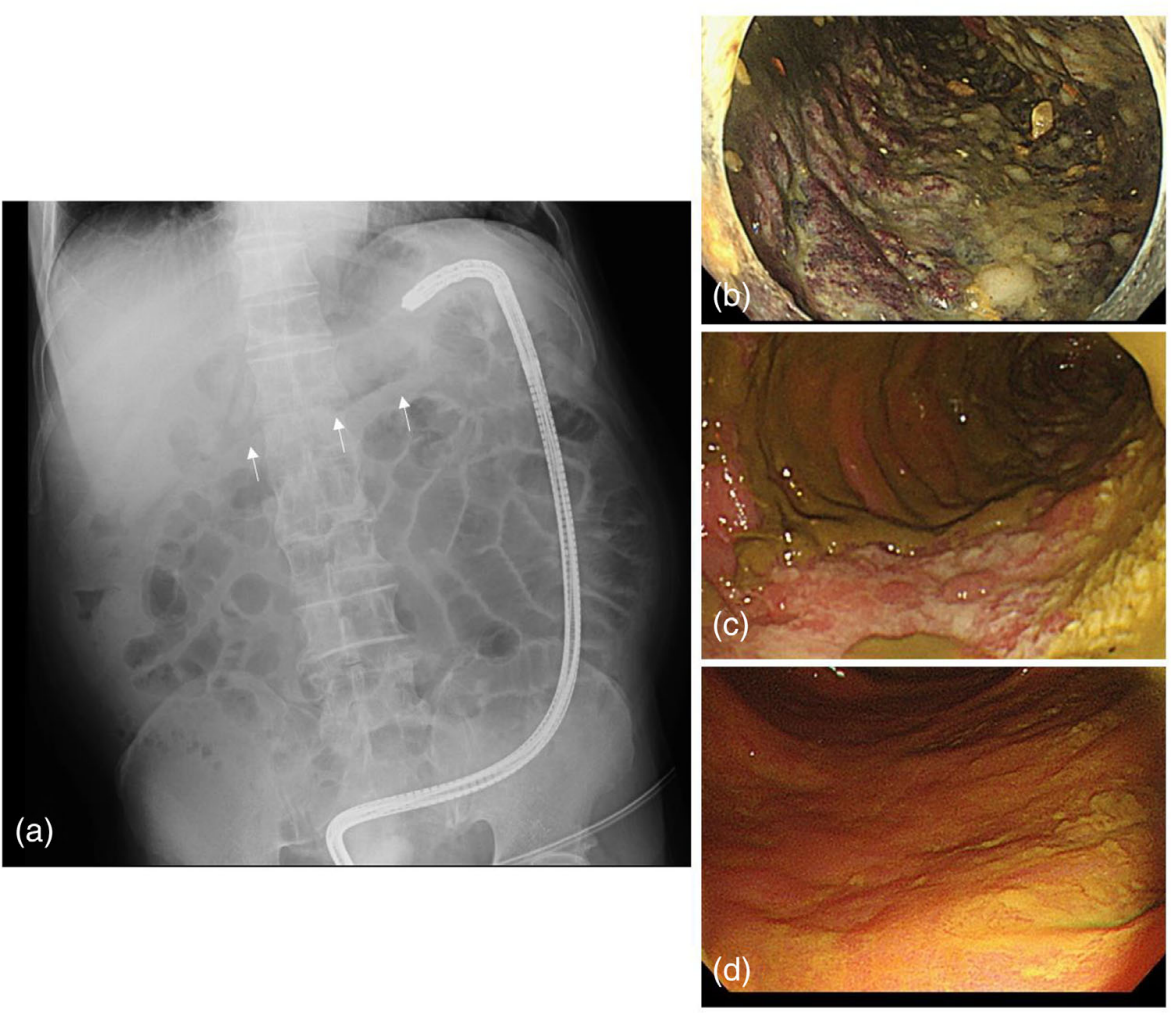
Endoscopic findings of obstructive colitis before/after endoscopic decompression. (a) Improved colonic dilation after endoscopic decompression. (b) Mucosa of the dilated colon appears black, suggesting severe ischemia (day 3). (c, d) Colonic mucosa gradually healed following multiple therapy sessions (days 18 and 30).

**FIGURE 3:**
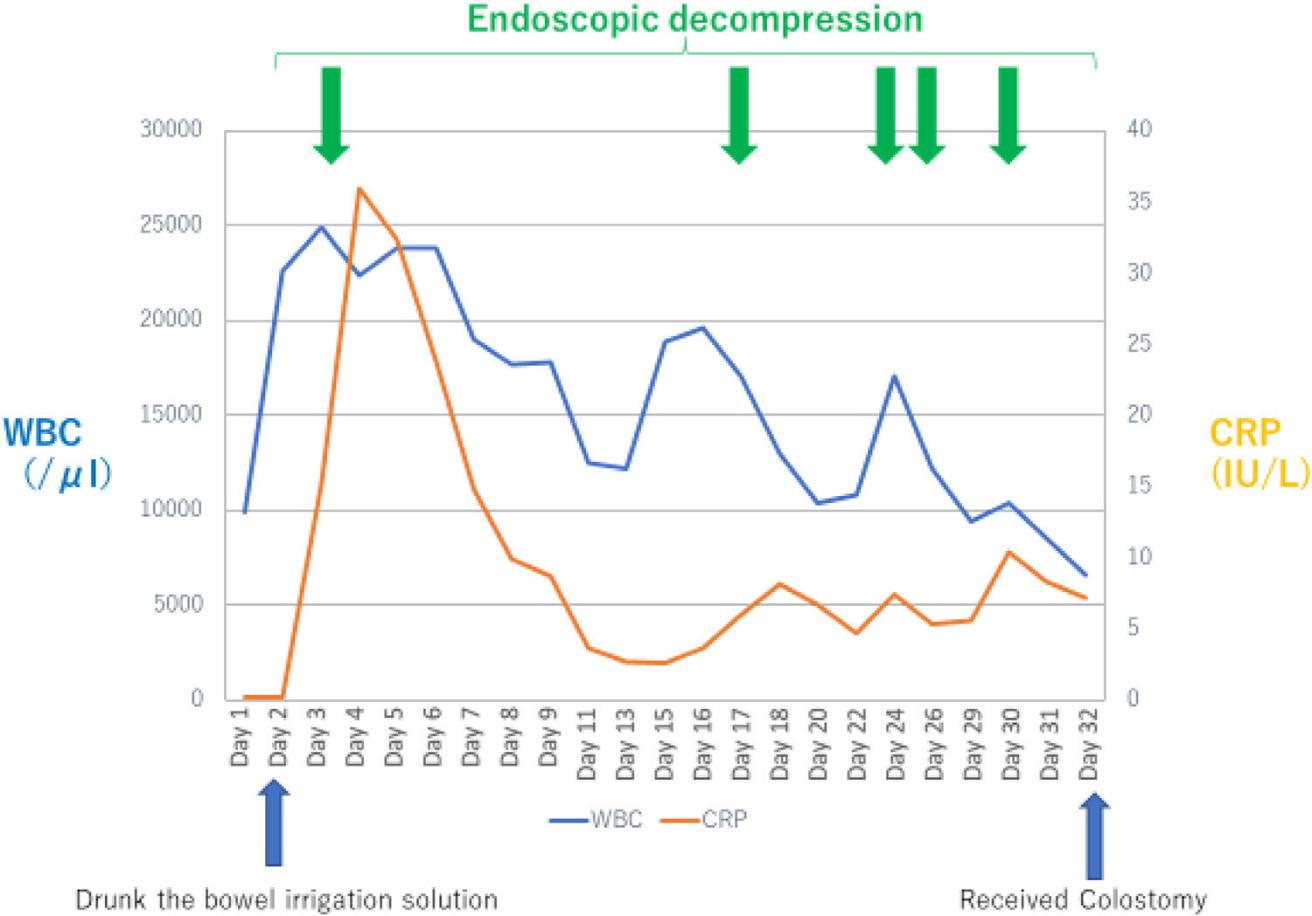
The clinical course of the patient.

After drinking the bowel irrigation solution, the patient developed severe abdominal pain and nausea. Laboratory data showed the following elevated values: White blood cell at 24,900, C‐reactive protein at 2.48 mg/dl, and lactate dehydrogenase at 337 IU/L. Tumor markers were at normal levels, with carcinoembryonic antigen at 4.5 ng/ml and carbohydrate antigen 19‐9 at 38.0 U/ml. Other laboratory data including creatine kinase (19 IU/L) and potassium (3.5 mEq/L) were also within normal ranges. Radiography and enhanced computed tomography demonstrated colonic dilation with maintained blood flow in the deeper layer of the colonic wall, which was caused by obstruction owing to a stricture with diverticulosis at the sigmoid colon (Figure 1a–c). As the colonic dilation in the cecum was very severe (>10 cm; Figure 1d), we immediately performed endoscopic decompression to avoid perforation. CO_2_ was emitted during the colonoscopic procedure in order to minimize the amount of air. The severe dilation of the colon improved successfully (Figure 2a); however, the mucosa of the dilated colon from the right half of the transverse colon to descending colon appeared black, suggesting severe ischemia (Figure 2b). Emergent colostomy for decompression of colonic dilation could not be performed safely for the wide range of severe colitis. Surgical total colectomy was considered owing to extensive colitis, from the cecum to the sigmoid colon. However, considering that blood flow was maintained in the colonic wall, we adopted a conservative approach after obtaining informed consent from the patient and consulting with surgeons. Relapse of colonic dilation occurred several times, accompanied by high fever and elevated inflammatory markers in the blood (maximum WBC level: 19,600 [day16] and maximum C‐reactive protein level: 8.18 [day18]), suggesting a toxic megacolon. Antibiotic treatment and repeated endoscopic decompression (five times; day 3, day 18, day 24, day 26, and day 30) successfully suppressed the colonic dilation and systemic inflammation (Figure 3). The colonic mucosa healed gradually following multiple therapy sessions for several weeks (Figure 2c,d), and we performed a colostomy without resecting a large part of the colorectum. After the operation, the patient was able to eat food orally without recurrent colitis and was discharged. We plan to evaluate the colorectal strictures with colonoscopy and a radiographic contrast enema, determine the extensiveness of the resected colon, and perform a colectomy and colostomy closure in the future.

## DISCUSSION

Obstructive colitis develops in the colon proximal to benign or malignant obstructions, most commonly in the rectosigmoid colon. Approximately 30% of obstructive colitis cases are caused by colorectal diverticulosis. Previous studies have shown that 1%−7% of patients with a colonic obstruction developed obstructive colitis. This type of colitis is induced by ischemia due to high endoluminal pressure and distension of the colonic wall.^6^ A rapid elevation of endoluminal pressure leads to massive dilatation, with a stretched bowel wall, necrosis, and sometimes perforation, similar to the characteristics of toxic megacolon. In particular, retention of bowel irrigation solution due to colonic obstruction often causes severe outcomes due to its unabsorbed nature. Thus, immediate decompression by either surgical or endoscopic approach should be considered to avoid necrosis of the entire colonic wall. In the current case, endoscopic deaeration and drainage of bowel irrigation solution might have improved the pathogenesis by lowering the endoluminal pressure.

According to several previous reports, severe obstructive colitis cases with black mucosa generally require surgical resections for a wide portion of the colorectum.^1^, ^4^ However, this case demonstrates that endoscopic decompression is optional if an enhanced computed tomography scan shows maintained blood flow. Moreover, no peritoneal irritation sign, and no elevation of deviation enzymes such as lactate dehydrogenase, creatine kinase, and potassium are additional criteria for this conservative therapy. Given that almost all colectomies worsen the quality of life of patients, endoscopic deaeration may be a candidate strategy for patients with severe obstructive colitis.

Regarding gastrointestinal decompression, an ileus tube and metallic stent might be the candidate therapy for the current case. However, the peroral ileus tube could not immediately decompress the dilation at the cecum. Moreover, the transanal ileus tube was considered to be at risk for perforation due to the severe mucosal damage of the dilated colon. A metallic stent could not be used for benign intestinal stenosis in Japan.

In conclusion, severe obstructive colitis with maintained blood flow can be treated with endoscopic decompression instead of emergent resection for a wide portion of the colorectum. Moreover, endoscopic images of improved colitic mucosa obtained through repeated colorectal procedures are rare and noteworthy.

## CONFLICT OF INTEREST STATEMENT

None.

## ETHICS STATEMENT

Written informed consent was obtained from the patient for the publication of this case report and accompanying images. A copy of the written consent is available for review by the Editor‐in‐Chief of this journal.

## Data Availability

The datasets used and analyzed during this study are available from the corresponding author upon reasonable request.
